# Changes in CD4^+^ T cells subsets in patients with alcohol-related cirrhosis

**DOI:** 10.1007/s10238-024-01548-0

**Published:** 2025-01-05

**Authors:** Paola Zuluaga, Coral Zurera-Egea, Daniel Fuster, Anna Hernandez-Rubio, Aina Teniente-Serra, Eva Martínez-Cáceres, Roberto Muga

**Affiliations:** 1 Universitat Autònoma de Barcelona, Bellaterra, Spain; 2 Department of Internal Medicine, Hospital Universitari Germans Trias I Pujol, Ctra. Canyet, Badalona, Spain; 3 Department of Cell Biology, Physiology and Immunology, Universitat Autónoma de Barcelona, Bellaterra, Spain; 4 Department of Immunology, Hospital Universitari Germans Trias I Pujol, Badalona, Spain

**Keywords:** T cell differentiation, T cell subsets, Adaptive immunity, Alcohol use disorder, Alcohol-related cirrhosis

## Abstract

Alcohol-related cirrhosis (AC) is a condition that impacts in immunity. We analyzed changes over time in CD4^+^subsets in AC-patients. We included patients with alcohol use disorder admitted at least twice for treatment. Patients without evidence of liver disease were the control group (CG). We analyzed naïve, memory, TEMRA, Th1, Th2, Th17, early activated, and late activated subsets. During the follow-up, TEMRA T cells were increased (1.2 ± 1.1% vs. 2.1 ± 1.1%, *p* = 0.03) in AC-patients (*n* = 5) and Th17 cells were decreased (14.1 ± 4.3% vs. 12.3 ± 6.4%, *p* = 0.03) in the CG (*n* = 22). Late activated cells were associated with a decrease in memory cells in both the groups. This association was stronger in AC-patients (*r* =  − 0.90, *p* = 0.04). The proportion of memory cells was correlated with an increased of Th1/Th2/Th17 cells  in the CG (*r* = 0.70, *r* = 0.68, *r* = 0.63; *p* < 0.01, respectively), whereas in AC-patients was correlated with a decrease in Th17 cells (*r* =  − 0.90, *p* = 0.03). AC-patients showed an increase in the proportion of TEMRA T cells, loss of heterogeneity and decreased CD4^+^ differentiation.

## Introduction

CD4^+^ T cells are heterogeneous in terms of antigen specificity, proliferative capacity, and the production of cytokines and effector molecules [1]. After activation, CD4^+^ T cells differentiate into central and effector memory cells, which recirculate through blood and lymphoid organs. Depending on the external signals received by the T cell receptor and the mitochondrial energy metabolism [2], central and effector memory cells may differentiate into different subsets, such as Th1, Th2, Th17, Th22, Th9, follicular Th, and T regulatory cells [3]. These subsets play a key role in directing the immune response against a variety of pathogens and are involved in a substantial number of inflammatory diseases.

Lymphocyte subsets are dynamic and may present mixed or intermediate phenotypes in response to changes in the molecular composition of the cellular environment. For example, Th17 cells have a high transformation capacity and show functional characteristics primarily associated with Th1 or Th2 cells [4].

Clinical and preclinical studies have shown that ethanol exposure alters mitochondrial function by increasing mitochondrial DNA damage and oxidative stress [5]. These changes affect the activation and differentiation capacity of lymphocytes, promoting their initial differentiation into pro-inflammatory Th1 cells [6]. Furthermore, patients with excessive alcohol consumption experience an increase in memory and effector lymphocyte subsets, an increase in the activation marker expression of CD38^+^ and HLA-DR^+^ cells, and a tendency to polarize into a Th2-related immune response. These changes promote chronic inflammation and inappropriate antigen-specific responses [7].

Alcohol-related liver disease (ARLD) alters cellular responses against pathogens [8]. The most studied form of ARLD is acute alcoholic hepatitis, which is characterized by a humoral inflammatory response and activation of T cells, favoring hepatocyte apoptosis and stimulating a fibrotic response [9]. However, despite the increase in activation markers in the acute phase of liver disease, patients with ARLD present with lymphopenia and a loss of naïve subsets [10].

This pilot study aimed to determine the changes in the differentiation and activation of CD4^+^ T cells and the association between their subsets in patients with alcohol-related cirrhosis (AC). Our hypothesis was that CD4^+^ T cell differentiation and activation are affected by the presence of advanced chronic liver disease in patients who continue to consume alcohol.

## Material and methods

### Patients

This was a cross sectional study involving a cohort of patients with alcohol use disorder (AUD) admitted to a detoxification program between January 2014 and October 2021. The patients were referred to primary care by an addiction specialist. The inclusion criteria for hospital detoxification were: a high risk of severe alcohol withdrawal (i.e., previous episodes of severe alcohol withdrawal and/or prior history of seizures or delirium tremens), the presence of medical comorbidities, and a history of outpatient detoxification failure. The exclusion criteria for hospital detoxification were severe psychiatric and/or acute comorbidities (i.e., acute infection or decompensated liver disease).

We included patients admitted at least twice for treatment and evaluated them for chronic alcohol liver disease. Patients without clinical evidence of liver disease according to ultrasound criteria and Fibrosis 4 (FIB-4) < 2.67 were used as the control group. The presence of liver cirrhosis was evaluated according to clinical and ultrasound criteria. Those with a history of infection with hepatitis C Virus (HCV), hepatitis B virus (HBV), or human immunodeficiency virus (HIV) were excluded.

On admission, the patients underwent anamnesis for clinical and AUD history, medical comorbidities, and physical examinations. Peripheral blood analyses were performed on the second day and abdominal ultrasonography on the third or fourth day. A physical examination was performed to evaluate the presence of jaundice, collateral circulation, abdominal ascites, and inferior limb edema. Blood tests included blood count, coagulation test, serology for HCV and HIV infection, HBV status, liver profile, and FIB-4 index assessment [11]. We performed abdominal ultrasonography at the Radiology Department under fasting conditions and assessed the presence and degree of liver steatosis and hepatomegaly, as well as signs suggestive of advanced chronic liver disease, such as heterogeneous liver, portal hypertension, ascites, and cirrhosis.

### Flow cytometry

We obtained 50 μL of fresh peripheral blood that were incubated with monoclonal antibodies for 20 min at room temperature and protected from light. Erythrocytes were removed by lysis (BD FACS™ Lysing Solution, Beckton Dickinson [BD] Biosciences, San Jose, CA, USA). The samples were then washed and resuspended in flow cytometry staining buffer (BD FACSFlow™, BD Biosciences). Cell subpopulations were studied using combinations of the following monoclonal antibodies: anti-CD3 V450, anti-CD4-PerCPCy5.5, anti-CD8 APCH7, anti-CD45RA PECy7, and anti − CCR7 PE (all from BD Biosciences), and anti-CD45 Alexa Fluor 700, anti-CXCR3 Alexa Fluor 488, and anti − CCR6 BV605 (from Biolegend, San Diego, CA, USA). A total of 10,000 lymphocytes were acquired and analyzed using a flow cytometer (LSRFortessa; BD Biosciences).

The lymphocyte population was gated by forward and side scatter, and the absolute count and relative percentage of populations of interest were determined. Absolute counts for each cell population were calculated as (X × Y)/100, where X is the percentage of each subset and Y is the absolute count of lymphocytes (Hematology Analyzer, Beckman Coulter, Miami, FL).

### Immunophenotyping of lymphocyte subsets

We determined the percentages and absolute numbers of T cells (CD3^+^), CD4^+^ T cells (CD3^+^ CD4^+^), and CD8^+^ T cells (CD3^+^ CD8^+^). Subsets of differentiation phenotypes included naive T cells (CCR7^+^/CD45RA^+^), central memory cells (CCR7^+^/CD45RA^−^), effector memory cells (CCR7^−^/CD45RA^−^), and terminal effector T cells (TEMRA) (CCR7^−^/CD45RA^+^). We divided CD4^+^ memory T cells into the following phenotypes: Th1 (CXCR3^+^/CCR6^−^), Th2 (CXCR3^−^/CCR6^−^), and Th17 (CXCR3^−^/CCR6^+^). To analyze memory T cells, we grouped the central and effector memory cells. Among total CD4^+^ T cells, we obtained the main activation phenotypes: early activated T cells (CD38^−^/HLA-DR^+^) and late activated T cells (CD38^+^/HLA-DR^−^). The subpopulation analysis followed the protocol of the European study ENTIRE Action [12, 13]. We expressed the absolute number of total lymphocytes as cell/μL.

**Fig. 1:**
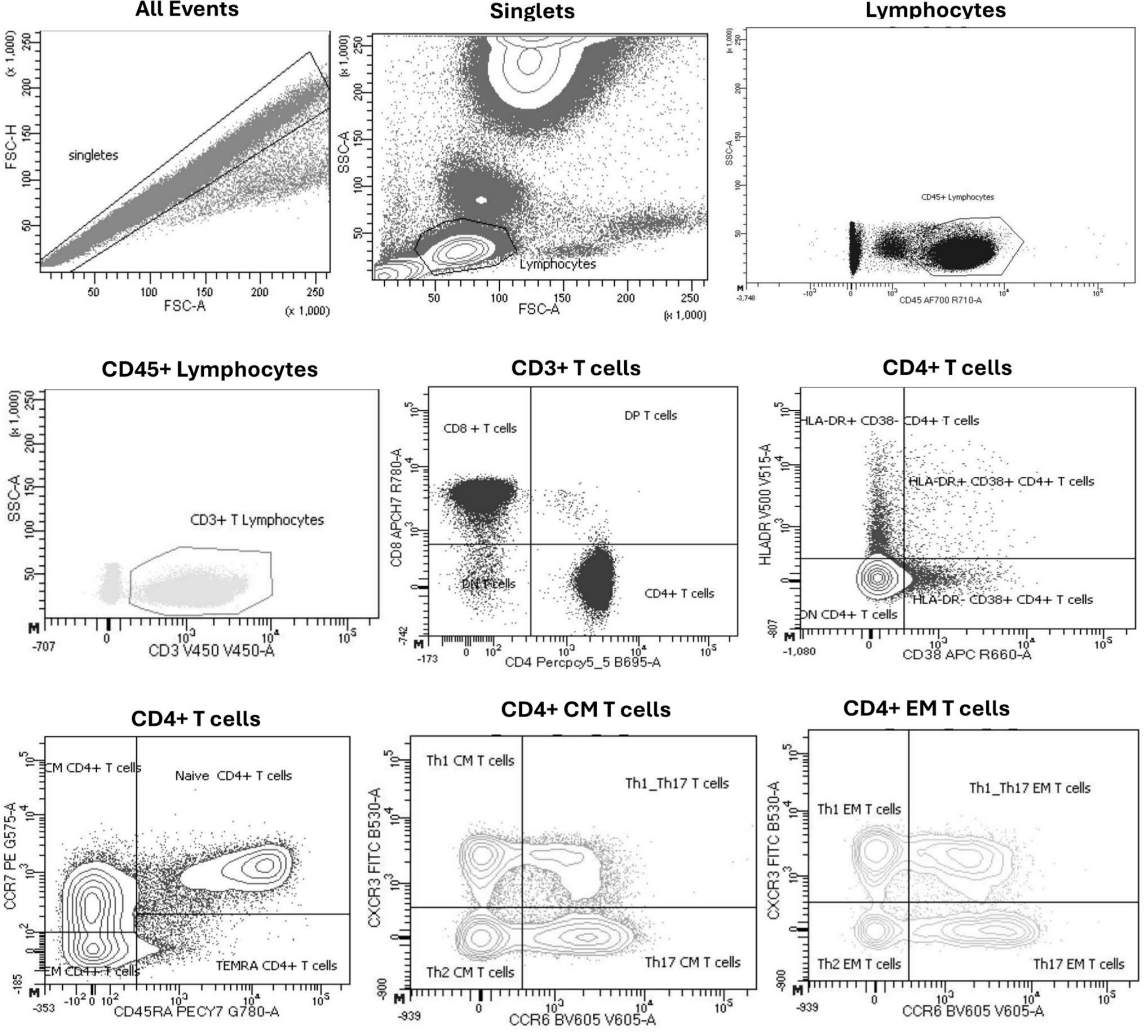
Cytometry strategy

The gating strategy applied to obtain the cell subsets does not include fluorescence minus one (FMO) controls as it is a well-established strategy and subsets are well defined. Our gating strategy is based on that applied at other Federation of Clinical Immunology Society Centers [14], which does not include FMO controls and it is also used as a standard practice for immune disorder diagnosis. In the Fig. 1 shows the cytometry strategy applied.

### Statistical analysis

Descriptive statistics were expressed as mean ± standard deviation (SD) or median (interquartile range [IQR]) for quantitative variables, and as absolute frequencies and percentage for qualitative variables. We compared lymphocyte phenotyping results between the initial admission and readmission periods for each patient. We performed a descriptive and paired analysis of each T cell subset and used Student’s T-test and Signrank test to detect significant differences, as appropriate. To highlight the relationship between subsets, we described the quotients of Th1 cells according to Th2 cells, Th1 cells according to Th17 cells, and Th2 cells according to Th17 cells. Values of p < 0.05 were considered statistically significant. Statistical analyses were performed using Stata software (version 11.1; College Station, Texas, USA).

## Results

### Laboratory data and lymphocyte subsets

We included 27 patients (81% men). The mean age was 46 ± 8 years, BMI was 25 ± 5.7 kg/m^2^, alcohol consumption was 186 ± 112 gr/day, and AUD duration was 17.4 ± 9.5 years. The average time between the first admission and readmission was 18 months [IQR: 9–29 months]. Five patients with AC were included in the study group, and twenty-two patients without liver disease were included in the control group. The control group showed a median FIB-4 index of 1.0 [IQR: 0.88–2.0] and 58% of these patients had liver steatosis as the only ultrasonography finding.

**Fig. 2:**
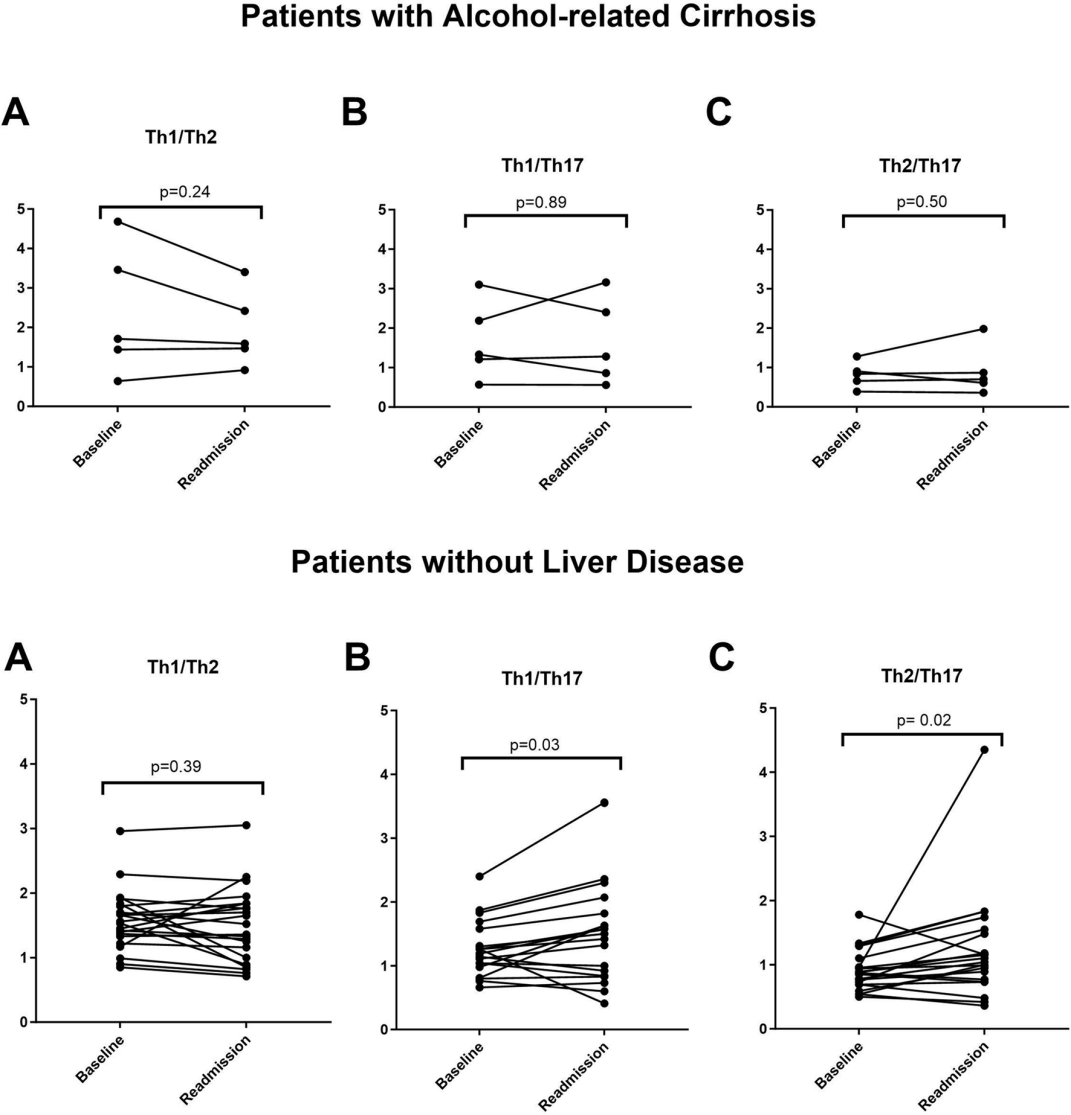
Differences observed in the quotient of **A** Th1/Th2 cells, **B** Th1/Th17 cells and **C** Th2/Th17 cells between during the follow-up in patients with alcohol-related cirrhosis and in patients without liver disease

Platelet and leukocyte counts, hemoglobin levels, and liver function were similar in both periods. Sociodemographic and laboratory parameters are shown in Table 1. The cell subsets were similar in both periods. However, during the follow-up there was an increase in TEMRA CD4^+^ T cells (1.24 ± 1.16% vs. 2.14 ± 1.14%, p = 0.03) in patients with AC, and a decrease of Th17 cells (14.16% ± 4.31 vs. 12.35% ± 6.40, p = 0.03) in patients without liver disease. Detailed information on the lymphocyte subsets is shown in Table 2. Moreover, an increase in the quotients of Th1/Th17 cells (1.44 ± 0.94 vs. 2.29 ± 2.7, *P* = 0.03) and Th2/Th17 cells (0.89 ± 0.31 vs. 1.65 ± 2.16, *p* = 0.02) were observed in the control group during the follow-up, as shown in Fig. 2. In these patients, a linear correlation was confirmed between Th1 and Th2 cells (*r* = 0.475, *p* = 0.02), and between Th2 and Th17 cells (*r* = 0.101, *p* = 0.65). In patients with AC, no significant variation was observed regarding the quotients of T-helper subsets: Th1/Th2 cells (2.3 ± 1.6 vs. 1.9 ± 0.9, *p* = 0.24), Th1/Th17 cells (1.6 ± 0.9 vs. 1.6 ± 1, *p* = 0.80) and Th2/Th17 cells (0.81 ± 0.32 vs. 0.90 ± 0.62, *p* = 0.50).

**Table 1:** Sociodemographic and laboratory characteristics in patients with alcohol-related cirrhosis and patients without chronic liver disease

	Alcohol-related liver cirrhosis n = 5			Patients without chronic liver disease n = 22		
	Baseline	Readmission	*p*	Baseline	Readmission	*P*
Age at admission	51 [50–54]	52 [50–56]	< 0.01	46 [39–41]	47.5 [41–52]	< 0.01
Males	4 (80)	–	–	18 (81)	–	–
BMI	23.3 [22.1–36.2]	25 [23.8–33.4]	0.62	22.7 [21–26.1]	22.9 [21–26.4]	0.46
Alcohol consumption (g/day)	140 [110–200]	250 [216–264]	0.16	**155 [120–200]**	**120 [100–168]**	**0.03**
Age of onset of alcohol consumption	16 [15–16]	–	–	17 [16–20]	–	–
Age of Alcohol Use Disorder	23.3 [15–30]	–	–	15.5 [9–20]	–	–
Hemoglobin (g/dL)	14.3 [13.7–14.7]	14.2 [13.8–14.8]	0.196	14.6 [12.7–15.2]	14.5 [13.4–15.5]	0.30
Leukocytes (× 10^9^/L)	4 [3.3–4.8]	3.7 [2.2–4]	0.248	6.6 [5.2–7.6]	6.4 [5.2–7.8]	0.41
Platelets (× 10^9^/L)	95 [52–102]	91 [63–127]	0.289	208.5 [157–239]	218.5 [176–287]	0.069
Albumin (g/dL)	38.6 [34–40.5]	38.1 [35.4–42.5]	0.445	40.3 [38.5–41.5]	39.1 [36.8–41.6]	0.233
Bilirubin (g/dL)	1.1 [1–2.28]	1 [0.8–1.47]	0.207	0.79 [0.52–1.1]	0.77 [0.52–1]	0.573
AST (U/L)	72 [40–149]	72 [39–84]	0.726	27 [20–44]	31 [20–50]	0.864
ALT (U/L)	47 [40–58]	43 [31–71]	0.732	32.5 [21–46]	36.5 [21–50]	0.589

**Table 2:** Main lymphocyte subsets in patients with alcohol-related liver cirrhosis and patients without chronic liver disease

	Alcohol-related liver cirrhosis n = 5			Patients without chronic liver disease n = 22		
%	Baseline	Readmission	*p*	Baseline	Readmission	*p*
T cells	77.7 ± 11.3	81.3 ± 4.6	0.89	77.55 ± 6.37	77.63 ± 8.33	0.96
CD4 T cells	48.8 ± 8.8	50.5 ± 9.3	0.68	48.03 ± 10	47.56 ± 9.80	0.80
CD8 T cells	25.1 ± 10.1	25.1 ± 6.8	0.99	25.76 ± 8.37	25.80 ± 9.44	0.97
CD4 Naive	19.9 ± 10.8	19 ± 8.25	0.81	34.60 ± 11.78	37.5 ± 13.84	0.27
CD4 Memory	78.8 ± 11	78.8 ± 8.7	1.00	61.2 ± 13.7	59.2 ± 16.8	0.30
CD4 TEMRA	**1.24 ± 1.16**	**2.14 ± 1.14**	**0.03**	3.60 ± 7.09	3.28 ± 5.65	0.67
Th1 cells	18.8 ± 7.5	18.5 ± 8.7	0.86	18.27 ± 6.32	19.05 ± 7.48	0.53
Th2 cells	10.8 ± 5.9	11.3 ± 6.6	0.65	12.0 ± 3.43	13.37 ± 5.10	0.10
Th17 cells	12.7 ± 4	13.3 ± 5.7	0.68	**14.16 ± 4.31**	**12.35 ± 6.40**	**0.03**
CD4 HLA-DR^−^/CD38^+^	18.5 ± 8.9	22 ± 14.1	0.49	26.2 ± 12.1	26 ± 11.3	0.92
CD4 HLA-DR^+^/CD38^−^	4.26 ± 1.45	5.06 ± 2.46	0.51	4.3 ± 2.99	3.18 ± 2.38	0.10

### Correlation between activated T cells and the proportion of memory T cells and TEMRA CD4^+^ T cells

**Fig. 3:**
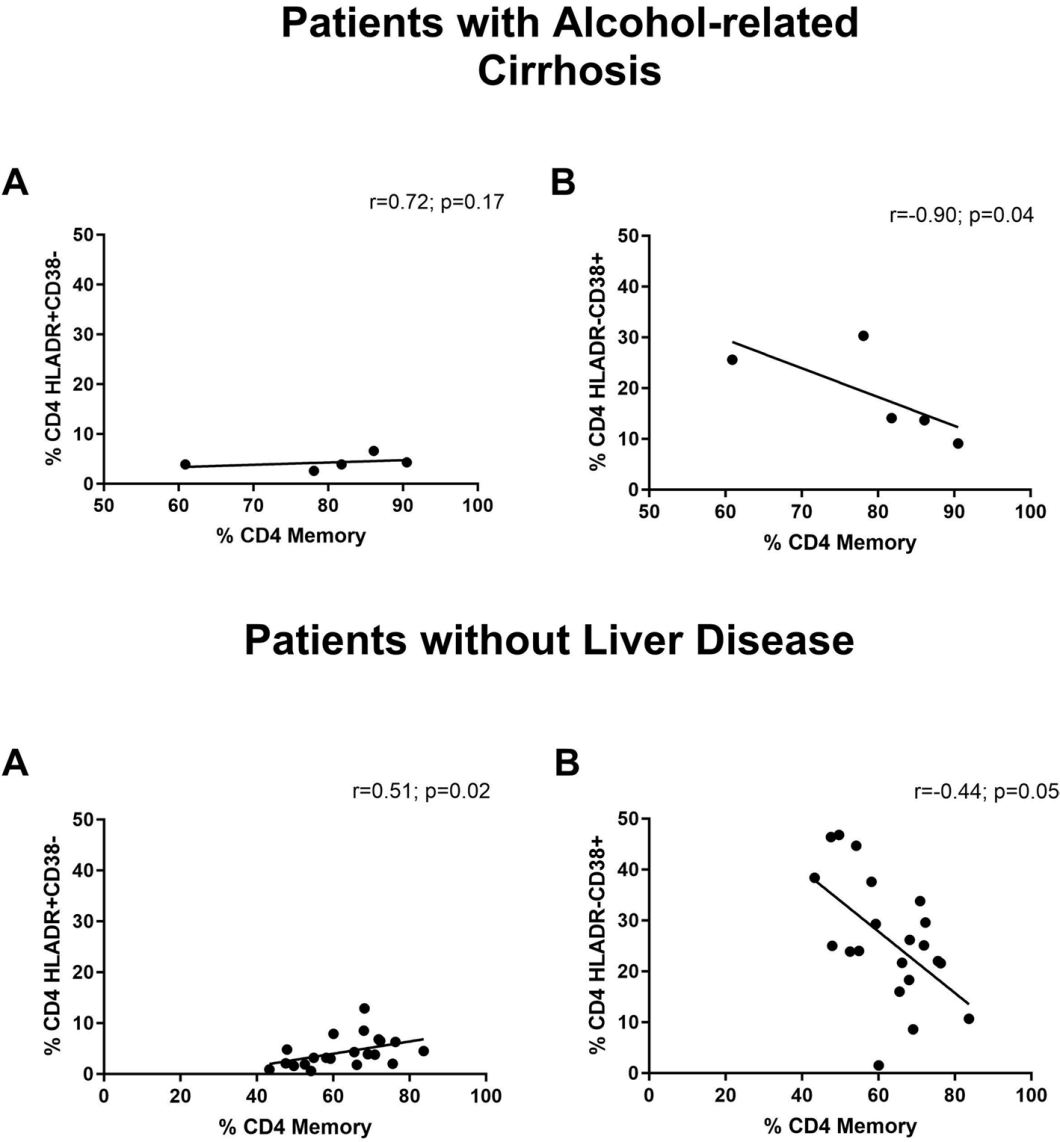
Correlations observed between memory CD4^+^ T cells with **A** early activated T cells (CD38^−^/HLA-DR^+^) and **B** late activated T cells (CD38^+^/HLA-DR^−^), in patients with alcohol-related cirrhosis and in patients without liver disease

As shown in Fig. 3, early activated T cells were related to an increased proportion of memory cells in the control group (*r* = 0.51, *p* = 0.02). However, late activated T cells were correlated with a decreased proportion of memory T cells in both groups. This correlation was stronger in patients with AC than in the control group (*r* =  − 0.90, *p* = 0.04; *r* =  − 0.44, *p* = 0.05, respectively).

There was no correlation between late or early activated T cells and the proportion of TEMRA CD4^+^ T cells in patients with AC (*r* = 0.526, *p* = 0.362; *r* =  − 0.102, *p* = 0.86) or in patients without liver disease (*r* =  − 0.148, *p* = 0.52; *r* =  − 0.177, *p* = 0.44, respectively). However, we found that the expression of CD38^+^ in CD8^+^ T cells correlated with an increased proportion of TEMRA CD4^+^ T cells in patients with AC (*r* = 0.872, *p* = 0.05).

### Correlation between the proportion of memory T cells and proportion of T-helper subsets

**Fig. 4:**
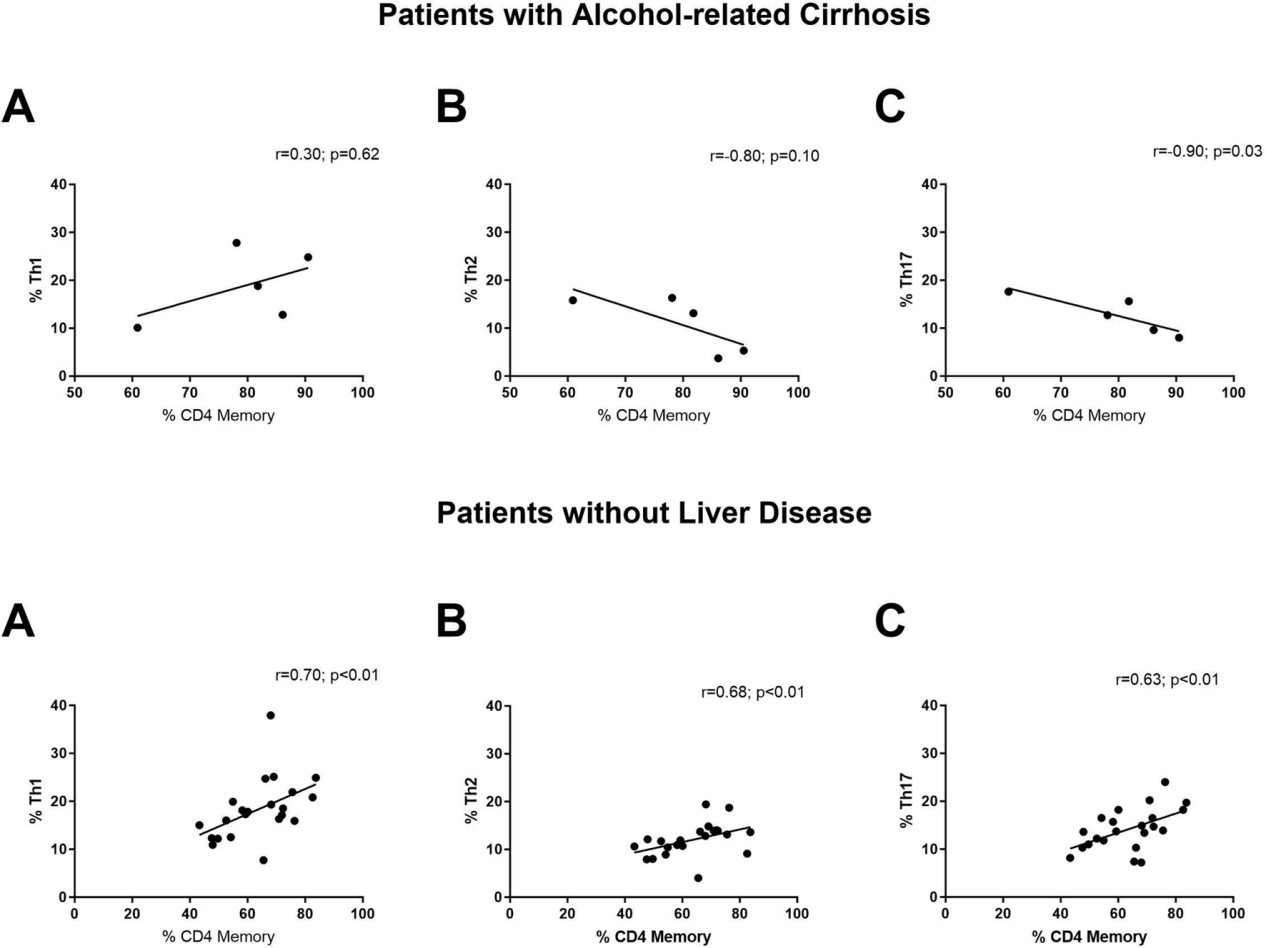
Correlations observed between CD4^+^ memory T cells with the percentage of **A** Th1 cells, **B** Th2 cells and **C** Th17 cells in patients with alcohol-related cirrhosis and in patients without liver disease

Figure 4 shows that the proportion of memory T cells was related to an increase in the proportion of Th1, Th2, and Th17 cells in the control group (*r* = 0.70, *r* = 0.68, *r* = 0.63; *p* < 0.01, respectively). In patients with AC, the proportion of memory cells was associated with a decrease in the number of Th17 cells (*r* =  − 0.90, *p* = 0.03).

## Discussion

This pilot study suggests an increase in TEMRA CD4^+^ T cells and a loss of cellular differentiation of CD4^+^ T cells in patients with alcohol-related cirrhosis who continue to drink alcohol.

TEMRA CD4^+^ T cells are heterogeneous subsets with increased expression of senescence markers and secretion of pro-inflammatory cytokines, chemokines, and proteases. This protein profiles characterize senescence-associated secretory phenotypes (SASP) [15, 16]. Moreover, TEMRA CD4^+^ T cells are associated with cytotoxic CD4^+^ T cells because they share genetic similarities [17]. Alterations in the function and integrity of the mitochondrial membrane have been described in these cells, which confer an inappropriate metabolic response after stimulation [18]. Despite the lack of a strong correlation between activated T cells and the proportion of TEMRA CD4^+^ T cells, CD8^+^ T cells expressing CD38^+^ were associated with an increase in TEMRA CD4^+^ T cells in patients with liver cirrhosis. Further research elaborating the relationship between TEMRA CD4^+^ T cells and cytotoxic populations could contribute to the description of the origin and the signals that favor the re-expression of the CD45 marker in T lymphocytes.

Although the proportion of memory cells during follow-up was similar in both groups, and the association between memory cells and activated T cells was similar in both groups, we found that patients with AC had association between late activated T cells and a decreased proportion of memory cells. Moreover, CD38 is an important regulator of lymphocyte functions and differentiation. Evidence suggests that its expression is related to the depletion of CD4^+^ T cells in HIV-1 infection [19], tumor progression in several oncologic diseases through the inhibition of CD4^+^ T cell differentiation into mature subsets, and tumor cell resistance to the inhibitory control of PD-1 [20, 21]. Therefore, determining the negative control exerted by the CD38 marker in CD4^+^ T cell differentiation and the mechanism by which this inhibition impacts the control of tumor cells and bacterial infections is critical in patients with liver cirrhosis.

In patients with no clinical evidence of chronic liver disease, alcohol consumption promotes cellular differentiation toward a Th1/Th2 response, with a decrease in Th17 cells. The heterogeneity of T-helper cells after cellular activation contributes to defining the types of immune and tissue responses to each type of pathogen. Th17 cells are characterized by plasticity, which promotes a polyvalent response, mainly in mucous membranes, against extracellular bacteria and fungi [22]. Furthermore, a dual role has been described for Th17 cells in alcohol-related liver diseases, favoring the recruitment of neutrophils to the liver through IL-17 [23] and simultaneously facilitating the regeneration of hepatocytes through IL-22 [24]. The expression of IL-22 by helper T cells (Th1, Th17, and Th22) has been associated with better prognosis in patients with acute alcohol-related liver disease [25].

In patients with liver cirrhosis, there was no correlation between memory cells and Th1 or Th2 cells. This loss of T cell differentiation might contribute to a lower capacity for antigenic recognition, favoring the risk and progression of bacterial infections [26], and thus, worsening the prognosis of liver disease [25].

To the best of our knowledge, this study is one of the first to approximate changes in CD4^+^ T cell subsets in patients with liver cirrhosis who continue to consume alcohol. However, the number of patients was a limitation, and the sample size must be expanded. Mechanistic studies are needed to describe the effect of alcohol on the mitochondrial dynamics of CD4^+^ T cells and the epigenetic effects at gene loci that regulate cellular plasticity in patients with different stages of alcohol-related liver disease. Finally, to understand the type of immune response in patients exhibiting an immune phenotype associated with immunosenescence is key to the design and development of immunomodulatory treatments in the chronic liver disease.

## Data Availability

The data used and analyzed during the current study are available from the corresponding author on reasonable request.
